# Evaluation of the Accumulation, Distribution, and Excretion of Different Silver Species in Tissues and Feces from Chickens and Pigs Fed with Silver-Based Nanomaterial Supplemented Feeds

**DOI:** 10.1021/acsagscitech.4c00338

**Published:** 2025-02-25

**Authors:** Khaoula Ben-Jeddou, Mariam Bakir, Maria S. Jimenez, Manuel Fondevila, Dino Metarapi, Martin Šala, Johannes T. van Elteren, Francisco Laborda

**Affiliations:** † Group of Analytical Spectroscopy and Sensors (GEAS), Institute of Environmental Sciences (IUCA), 16765 University of Zaragoza, Pedro Cerbuna, 12,50009 Zaragoza,Spain; ‡ Department of Animal Production and Food Science, University of Zaragoza,Miguel Servet 177,50013 Zaragoza,Spain; § Department of Analytical Chemistry, National Institute of Chemistry,Hajdrihova 19,1000 Ljubljana,Slovenia

**Keywords:** pigs and chickens, silver nanomaterial, accumulation, excretion, laser ablation-SP-ICP-MS

## Abstract

On the basis of two in vivo experiments involving pigs and chickens, where animals were fed with feeds supplemented with a silver–kaolin nanomaterial as a growth promoter, the fate of the ingested silver, its accumulation in different tissues and excretion, and its occurrence as ionic or particulate forms has been studied. Inductively coupled plasma mass spectrometry (ICP-MS) in conventional mode was used for the quantitative determination of total silver in the different tissues and feces. Results showed that silver accumulated in liver (0.15–5 mg kg^–1^) and kidney (0.4 mg kg^–1^) but not in muscle, while the major part of silver was eliminated through the feces (75–1026 mg kg^–1^). Laser ablation coupled to ICP-MS (LA-ICP-MS) in a conventional mode revealed a preferential accumulation of silver in the outer layer of the liver lobules in pigs. LA-ICP-MS in single particle mode (LA-SP-ICP-MS) was applied to the analysis of tissues and feces to obtain speciation information about the presence of ionic and particulate silver in the samples. Silver found in the pig liver was present in ionic form, confirming that silver was absorbed in the intestine in this form. The analysis of pig feces confirmed the presence of both ionic silver and particles containing silver with average masses of 520 ag (equivalent to solid spherical silver nanoparticles of 50 nm diameter).

## Graphical abstract

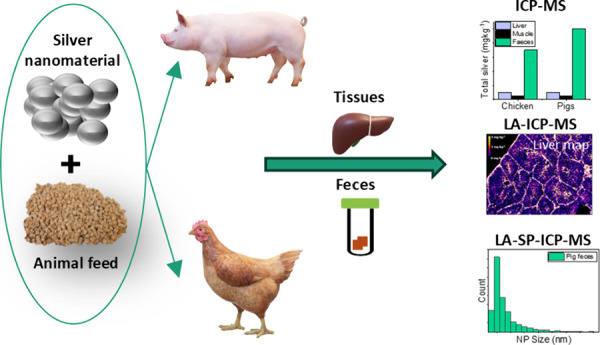

## 1 Introduction

Chicken and pork are the most consumed meats because of their lower price and healthier nutritional contribution.^1^ The use of antibiotics as feed additives once allowed one to increased livestock production by ensuring animal health but contributed to the emergence of antibiotic-resistant bacteria,^2–5^ which led to the banning of antibiotics as growth promoters in animal feed by the European Union in 2003.^6^ For this reason, alternative feed additives are being explored to replace antibiotics and to ensure healthy growth and safety of the animals.

Thanks to their antimicrobial properties,^7–9^ silver nanoparticles (AgNPs) are considered an alternative additive to conventional antibiotics in broiler^10–16^ and swine diets.^17–20^ In this line of action, AgNPs have demonstrated their biocidal effect against viruses, such as the African swine fever virus, which is fatal to pigs,^17^ and the Newcastle disease virus, which decreases poultry production by inhibiting viral cell entrance and virus replication.^21^ As antibacterial agent administrated to in vivo infected animals or to bacterial in vitro culture, AgNPs have decreased the growth of the antibiotic-resistant bacteria *Clostridium perfringens* biofilms in animals and humans^13,22^ and have been found to be an effective biocide against *Salmonella* strains.^23^ When supplied to feed at 20 and 40 mg of AgNPs for 35 days, AgNPs have increased pig daily growth compared to the control group and reduced ileal concentration of coliforms;^19^ thus, recently they have been considered a valuable growth promoter for chickens when added to poultry diet under a dose level of 4 mg kg^–1^ diet.^16^ AgNPs have also increased chickens’ body weight and muscle gain when fed basal diets supplemented with 50 mg kg^–1^ of Ag NPs for 12 days.^24^ When added to drinking water at 1 mM for 35 days, AgNPs have increased chicken productive performance and boosted their immune system,^25^ showing high antimicrobial activity against pathogens with an optimum dose of 50 mg L^–1^ for the disinfection test against total coliform bacteria in the farm water,^26^ reducing *Escherichia coli* presence and lowering chicken mortality rate.^27^

The European Food Safety Authority (EFSA), in its updated Guidance on Risk Assessment of Nanomaterials in the Food and Feed Chain and Human and Animal Health, outlined the importance of in vivo studies for the quantification of nanomaterials. The latter should be assessed in body tissues, fluids, and excreta; besides, its form should be characterized in biological samples.^28^

Although to date several in vivo studies have been performed on the fate of nanosilver uptake by livestock, silver has been mostly administered as pristine AgNPs and no differentiation between silver species has been carried out. The oral supplementation of 20 nm of AgNPs to chicken by gavage with a dose of 6 mg kg^–1^ for 22 days or the addition of 2.5–10 mg L^–1^ to water intake for 35 days^10,15^ revealed that silver was retained in the liver with an amount ranging from 141 to 269 μg kg^–1^ and increased when silver concentration increased, but it was not detected in tissues such as breast or muscles, whereas other studies have shown that after the inclusion of 2.5 to 20 mg kg^–1^ of AgNPs for 42 days, silver was accumulated in breast and thigh muscle,^12^ while a concentration of 4, 8, and 12 mg L^–1^ for 42 days^29,30^ and 15 mg L^–1^ for 40 days^29,30^ added to drinking water led to silver retention in the edible parts of chickens such as breast, femur, and liver with a value around of 0.1 mg kg^–1^ and a level of 0.04 and 0.08 mg kg^–1^ in kidney and liver tissues, respectively.

In most works, ICP-MS is used for the quantification of total silver in tissues, whereas in combination with laser ablation (LA-ICP-MS) information about the silver distribution can be obtained.^31^ Recently, LA-SP-ICP-MS has gained growing attention, and the concept and fundamentals of this novel methodology have been reported by Metarapi et al.^32–34^ For LA-ICP-MS operating in single particle mode, short dwell times in the microsecond range are recommended for the detection of individual nanoparticles and low laser fluences should also be used to prevent nanoparticle disintegration.^34^ Previous works involving the detection and characterization of AgNPs and Ag­(I) in biological samples using LA-SP-ICP-MS have been done using mouse tissues after intravenous or intraperitoneal injection of AgNPs,^35,36^ showing heterogeneous accumulation of Ag­(I) and AgNPs in liver and excretion through kidneys as Ag­(I). Nordhom et al.^37^ employed LA-SP-ICP-MS to confirm the translocation of intact AuNPs to the spleen after intratracheal instillation of the nanoparticles in rats.

The main objective of this study is the evaluation of the accumulation, distribution, and excretion of different silver species in tissues and feces from chickens and pigs fed feeds supplemented with a silver-based nanomaterial as a way to characterize the potential hazards that using silver in animal feeding may have on consumer health and on the environment. As a first step, the accumulation of silver in the different tissues and its excretion was studied by using conventional ICP-MS for the quantification of total silver. In a second step, a deeper insight about the location and the spatial distribution of silver species in pig and chicken tissues and feces was obtained using LA-ICP-MS in conventional and single particle detection modes. The novel approach LA-SP-ICP-MS has been applied for the imaging of Ag­(I) and AgNPs in lyophilized sections of liver and feces of the animals under study. To the best of our knowledge, the analysis of biological samples by LA-SP-ICP-MS after the administration of a silver-based nanomaterial in the diet had not been studied before, being relevant to explore the fate of silver nanomaterials after oral ingestion, gastrointestinal transformations, and elimination.

## 2 Materials and Methods

### 2.1 Procedures

#### 2.1.1 In Vivo Experiments

Two experiments were carried out using the Ag–kaolin nanomaterial as a supplement in the feed of chickens^40^ and pigs. Management and sampling procedures of the animals were approved by the Ethics Committee for Animal Experimentation of the University of Zaragoza (PI55/18). Feed and water were offered ad libitum. A total of 870 chickens at hatching were randomly allocated to three experimental treatments based on the pattern of silver nanoparticles (Ag–kaolin at 0.2% (m/m), 17 mg of Ag kg^–1^) administration: fed without (C1) or with silver nanoparticles for 21 (C2) or 35 (C3) days. All groups received the same feed without Ag–kaolin in the sixth experimental week. Birds were housed in 12 pens of 24 animals per treatment. At the end of the production period (42 days of age), 20 chickens per treatment were slaughtered for sampling.

For the other experiment, 60 weaned piglets (24 days of age) housed in 15 pens of 4 piglets each were randomly allocated to three treatments (20 piglets per treatment) based on the dose of silver nanoparticles (Ag–kaolin): without (P1) or with 20 (P2) or 200 (P3) of Ag–kaolin. Prestarter (from 0 to 14 days) and starter (from 15 to 35 days) compound feeds were made up with the corresponding doses of Ag–kaolin and offered ad libitum through the transition phase. At the end of the experiments (piglets of 63 days of age, after 49 days of experiment), 12 animals from P1, 17 from P2, and 18 from P3 were randomly chosen for slaughter and sampling. In both experiments, treatments without Ag–kaolin (C1 and P1) were considered as a control to avoid any side effects.

The sacrifice of the animals was done after 3 h of water and feed deprivation by stunning them with CO_2_. After being slaughtered, liver, muscle, kidney (only for pigs), and excreta (obtained directly from the rectum) were collected, conserved at −20 °C and lyophilized prior to analysis. Details of the experimental designs are included in the Supporting Information. Muscle parts chosen for sampling are representatives of the quality parts of pigs (ham; biceps femoralis, semitendinosus, and semimembranosus muscles) and poultry (breast; pectoralis major muscle) carcasses.

#### 2.1.2 Sample Collection and Preparation

At the end of the two in vivo experiments, samples from muscle, liver, kidney, and feces of 47 pigs from a total of 60 pigs, as well as from muscle, liver, and feces of 60 chickens from a total of 870 chickens (see Table S1 of the Supporting Information (SI)) were collected and lyophilized.

The LA-ICP-MS samples were kept whole for sectioning, whereas for totals, a part of the sample was ground. For acid digestion, tissues and feces were ground using a Retsch MM400 mixer mill (Retsch, Dusseldorf, Germany). A 2 g amount of the lyophilized samples was weighed and ground using stainless steel jars containing a 25 mm diameter ball (5 min for organs and 3 min for feces) at 25 Hz.

For LA-ICP-MS analysis, samples (see Table S2 of the SI) were embedded in paraffin and cryo-sectioned into thin slices of 2.5 and 5 μm using a Leica RM2255 rotary microtome prior to laser ablation analysis.

#### 2.1.3 Acid Digestion of Tissues and Feces

A 100 mg amount of the ground sample was weighed into a microwave digestion vessel. A 3 mL aliquot of HCl and 7 mL of HNO_3_ were added, and the digestion was performed at 200 °C and 800 psi for 30 min in a microwave oven (Mars 6 CEM, Charlotte, NC, USA). After digestion, the volume was made up to 50 mL with 3% (v/v) HCl.

#### 2.1.4 Gelatin Standards Preparation for Laser Ablation Analysis

Gelatin standards were prepared following the method of Šala et al.^41^ Gelatin was used to match biological matrices since it has been proven to provide results similar to matrix-matched on-tissue quantification.^42^ For LA-SP-ICP-MS analysis, homogeneous AgNPs and Ag­(I) standards were prepared by suspending the commercial standards in 10% (m/v) gelatin. A 50 mg amount of gelatin was weighted in Eppendorf tubes, and 400 μL of ultrapure water was added and dissolved at 55 °C until a clear solution was obtained. Subsequently, 50 μL of 20 nm (3.50 × 10^12^ nanoparticles mL^–1^), 40 nm (5.40 × 10^12^ nanoparticles mL^–1^), 50 nm (2.90 × 10^12^ nanoparticles mL^–1^) and 60 nm (1.70 x10^12^ nanoparticles mL^–1^) AgNPs and 5 μL of 10 nm (3.50 × 10^12^ nanoparticles mL^–1^) AgNPs were added. The final nanoparticle number concentration in these suspensions was 3.50 × 10^11^ nanoparticles g^–1^ for 10 and 20 nm, 5.40 × 10^11^, 2.90 × 10^11^, 1.70 × 10^11^ nanoparticles g^–1^ for 40, 50, and 60 nm AgNPs, respectively. Suspensions were thoroughly mixed until homogenization. For Ag­(I), two calibration curves were prepared, one for liver samples with low concentrations ranging from 0.5 to 4 mg kg^–1^ and another one for feces samples with higher concentrations of 20, 50, and 100 mg kg^–1^. A 1 g amount of gelatin was weighed in 15 mL tubes, and the corresponding volumes of Ag­(I) standards were added. Ultrapure water was added up to 10 mL and then dissolved at 55 °C. On a hot glass microscope slide, drops of each standard were deposited carefully, and glass slides were dried for 1 h at 100 °C in a convection oven.

#### 2.1.5 Distribution of Silver by Laser Ablation–ICP MS

Using LA-ICP-MS in continuous line scanning mode, the distribution of total silver concentration in the samples was determined using gelatin standards containing different concentrations of ionic silver. Raw data from the line scans were processed using ImageJ to obtain silver concentration maps.

#### 2.1.6 LA-SP-ICP-MS Data Processing

The data processing of AgNPs measurement was done as described in a previous publication using a custom script developed with MatLab R2020a (MathWorks).^33^ Silver mass per particle information was obtained as equivalent diameters by calibration with AgNPs standards prepared in gelatin, as described in Section 2.1.4 to simulate animal tissues.^41,42^ An in-house-developed MatLab script developed by Metarapi et al.^33^ was used to discriminate nanoparticle signals from the baseline and to obtain the signal distributions of the different AgNP standards in order to obtain the corresponding size calibration (Figure S1 of the SI). A discrimination threshold of 5 counts was considered, which corresponds to a size detection limit of 26 nm for solid spherical silver nanoparticles, equivalent to particles containing 96 ag of silver

### 2.2 Instrumentation

In order to determine the total amount of silver in tissues and feces, the digested samples were analyzed by ICP-MS. The instrument used was a PerkinElmer NexION 2000 ICP-MS (PerkinElmer, USA). The instrumental parameters are presented in Table S3 of the SI. Optimization of the instrument was carried out daily using a standard solution containing 1 μg L^–1^ of Be, Ce, Fe, In, Li, Mg, Pb, and U (in 1% HNO_3_ (v/v)) from the instrument manufacturer (PerkinElmer). Maximum silver sensitivity was achieved by optimization of the nebulizer gas flow and the lens voltage using a 1 μg L^–1^ Ag­(I) solution.

For the imaging of the different silver species in tissues and feces, the laser ablation system used throughout this work was an Analyte G2, 193 nm ArF* (Teledyne Photon Machines Inc., Bozeman, MT, USA) equipped with a two-volume ablation cell (HelEx II). The line scan routine was used to ablate the gelatin standards as well as the tissues and feces of pigs and chickens. The instrumental and acquisition conditions for LA-ICP-MS and LA-SP-ICP-MS are listed in Table 1. Origin (OriginPro 2018, OriginLab Corp., Northampton, MA, USA) was used for data processing. ImageJ was used for the manipulation of laser ablations maps.

**Table 1:** Laser Ablation and ICP-MS Parameters in Conventional and Single Particle Modes

	conventional mode	single particle mode
Laser Ablation Parameters		
He gas flow rate		
cup, L min^–1^	0.3	0.5
cell, L min^–1^	0.3	0.3
laser fluence, J cm^–1^	1	1
laser beam size, μm	10	20
dosage	10	10
repetition rate, Hz	200	4
scan speed, mm s^–1^	200	8
ablation mode	line	line
ICP-MS Parameters		
RF power, W	1500	
plasma gas flow, L min^–1^	15	
auxiliary gas flow, L min^–1^	0.9	
dwell time	50 ms	100 μs
isotope monitored	^107^Ag	

### 2.3 Chemicals

Silver­(I) standard stock solution of 994 ± 3 mg L^–1^ (Panreac, Barcelona, Spain) was used to prepare aqueous silver solutions by accurately weighing (±0.1 mg). Five 20 mg L^–1^ citrate-stabilized suspensions of silver nanoparticles of 10.3 ± 2.1, 20.8 ± 3.0, 41.5 ± 5, 50 ± 4, and 60 ± 7 nm diameters were purchased from NanoComposix (San Diego, CA, USA). Rhodium (Rh) standard stock solution of 1002 ± 6 mg L^–1^ (Sigma-Aldrich, St. Louis, MO, USA) was used as the internal standard for ICP-MS. Certified Dogfish Liver Reference Material DOLT-4 (*n* = 10) (National Research Council Canada, Ottawa, Canada) containing a certified silver amount of 0.93 ± 0.07 mg kg^–1^ was used to validate the accuracy of the total silver determination by acid digestion. Gelatin (porcine-skin gelatin, type A; bloom strength, 300; Sigma- Aldrich) was used for the fabrication of LA matrix-matched standards. For acid digestion and total silver content determination, nitric acid (69/70%, J.T. Baker, Phillipsburg, New Jersey, USA) and hydrochloric acid (36.5/38%, J.T. Baker, Phillipsburg, New Jersey, USA) were used. For the preparation of solutions, ultrapure water (resistivity, 418 MΩ cm^–1^) was collected from a Milli-Q Advantage system (Millipore, Billerica, MA, USA).

The nanomaterial involved in the present work (Ag–kaolin) consisted of AgNPs deposited on kaolin microparticles and had been characterized previously.^38^ The silver content in the formulation was 0.83 ± 0.04% (m/m) with spheroidal silver nanoparticles with diameters ranging from 2 to 90 nm and an average diameter of 27 nm. An in vitro genotoxicity study conducted by Rodriguez-Garraus et al.^38^ evaluated the toxicity of the nanomaterial on mouse cells, confirming no gene mutations or DNA aberrations. Furthermore, the antibacterial activity of the nanomaterial on a variety of bacterial strains was tested by Pérez-Etayo et al.,^39^ showing inhibition of different Gram-negative and Gram-positive bacteria.

## 3 Results and Discussion

### 3.1 Total Silver Content in Tissues and Feces of Pigs and Chickens by ICP-MS

The total content of silver in tissues and feces was quantified as described in Section 2.1.3. The analytical performance of the method was tested through the analysis of the certified reference material DOLT-4 (*n* = 10), obtaining a recovery of 101 ± 1%. The recovery of the method was also tested by using spiked control samples, obtaining recoveries in the range of 92–103%. The detection (LOD) and quantification limits (LOQ), calculated from 3 and 10 times the standard deviation of 10 blanks, were 0.03 and 0.1 mg kg^–1^, respectively.

The results for the pig and chicken samples are shown in Table 2. Regarding the analysis of tissues, the silver contents of control samples from animals nonfed with silver-supplemented feed, were below the LOQ. For the different feeding treatments tested, silver accumulation in muscle was not observed for both animals. However, there was a relevant accumulation of silver in pig liver (0.60 mg kg^–1^), when pigs were administered feed supplemented with 0.2% of the nanomaterial, which increased (4.71 mg kg^–1^) when pigs received feed containing 2% of Ag–kaolin, suggesting a proportional relation dose–accumulation. For chickens, results showed that silver also accumulated in the liver (0.60 mg kg^–1^) after 21 days of being fed with supplemented feed, although no silver was detected after 21 additional days of administering the control feed. The silver present in livers of chicken fed with supplemented feed for 35 days following 7 days with control feed also showed reduced levels of silver (0.15 mg kg^–1^), which outlines the relevance of a clearance period to eliminate residual silver from the organ. Accumulation of silver in the kidneys was also observed in pigs fed with feed supplemented at 2% Ag–kaolin (0.38 mg kg^–1^). These results are in agreement with previous studies involving oral administration of silver nanomaterials, where silver accumulation was higher in liver,^10,15,18,43^ followed by kidney^43^ or spleen.^44^

**Table 2:** Total Silver Content (mg kg^–1^) in Tissues and Feces of Pigs and Chickens by ICP-MS^a^

	pig		chicken	
sample	treatment	Ag content mg kg^–1^	treatment	Ag content mg kg^–1^
muscle	control	<LOQ	control	<LOQ
	P1	<LOQ	C1	<LOQ
	P2	<LOQ	C2	<LOQ
			C3	<LOQ
kidney	control	<LOQ	–	–
	P1	<LOQ	–	–
	P2	0.38 ± 0.1		
liver	control	<LOQ	control	<LOQ
	P1	0.60 ± 0.25	C1	0.60 ± 0.23
	P2	4.71 ± 2.11	C2	<LOQ
			C3	0.15 ± 0.08
feces	control	0.70 ± 0.26	control	0.27 ± 0.18
	P1	155 ± 30	C1	75 ± 12
	P2	1026 ± 178	C2	1.23 ± 0.33
			C3	2.0 ± 1.3

a Average ± standard deviation (*n* = 3). LOQ, 0.1 mg kg^–1^.

With respect to the analyses of feces, both animals showed significant levels of silver when they were continuously fed with a diet containing 0.2% Ag–kaolin (155 and 75 mg kg^–1^ for pigs and chickens, respectively). For pigs, this content increased almost 7 times, up to 1026 mg kg^–1^ for diets supplemented with 2% of additive. As expected, the content of silver in feces of chickens subjected to a clearance period of 7 or 21 days decreased to 2.0 and 1.2 mg kg^–1^, respectively.

The non-accumulation of silver in muscle decreases the potential exposure to the nanomaterial. Moreover, no toxicity signs were observed for chickens and pigs using concentration levels of 0.2 and 2% of the additive, although the dose of 0.2% would be more convenient to reduce silver accumulation in liver and kidneys. In a previous study, levels of 20–40 mg kg^–1^ of AgNPs were tested by Fondevila et al.;^20^ the authors reported that such doses promoted physiological and productive effects in animals without causing any toxicity or side effects. The chicken experiment revealed the importance of suppressing the nanomaterial prior to animal sacrifice to ensure the non-accumulation of silver in animal tissues.

### 3.2 Distribution of Silver in Tissues and Feces by LA-ICP-MS

The experimental conditions for the detection of silver in liver and feces by LA-ICP-MS in conventional mode are summarized in Table 1. A fluence of 1 J cm^–2^ was selected to prevent nanoparticle disintegration.^34^ Ten laser shots per pixel on sample slices of 5 μm thickness allowed the acquisition of a high silver intensity signal and good image resolution, as it is shown in Figure S2 of the SI. Other operational and computational LA-ICP-MS mapping conditions were selected from previous mapping studies.^45^

For total silver determination, gelatin standards with different concentrations of Ag­(I) were measured under the same conditions as the samples. A calibration from 1 to 4 mg kg^–1^ was used for liver samples, whereas a higher range from 20 to 100 mg kg^–1^ was selected for feces. Limits of detection and quantification, calculated as 3 and 10 times the standard deviation of the gas blank signal divided by the slope of the calibration curve, were 0.13 and 0.46 mg kg^–1^, respectively.

Figure 1 shows the distribution of silver in three liver slices from different pigs in group P2 fed with a diet containing 2% of the silver additive. It can be observed that silver was concentrated in the connective tissue surrounding the hepatic lobules.^46^ Samples from chickens and pigs fed with lower contents of the additive are not shown, since the accumulated silver was below the LOQ. Figure 2 shows the distribution of silver in feces from pigs fed diets containing 0.2 (P1) and 2% (P2) of the silver additive, confirming the higher concentration of silver as well as its homogeneous distribution across the sections analyzed.

**Figure 1:**
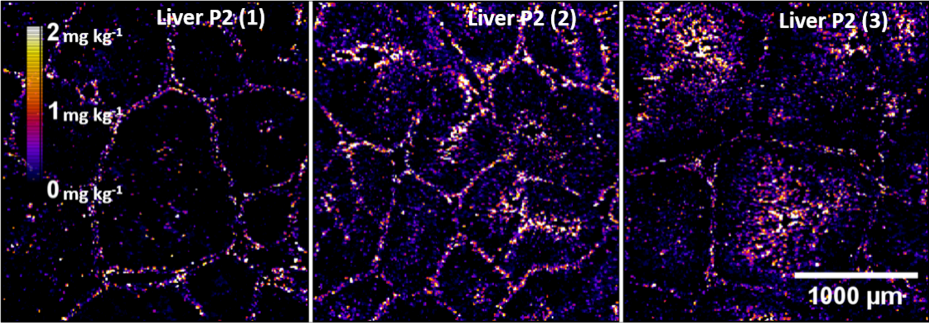
Total silver maps of pig livers from group P2 measured by LA-ICP-MS.

**Figure 2:**
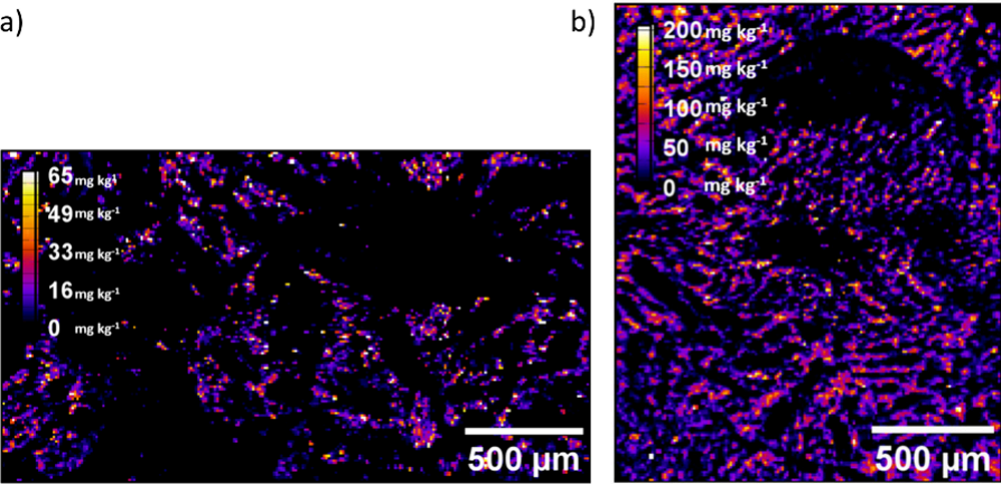
Total silver maps of pig feces from P1 (a) and P2 (b) groups measured by LA-ICP-MS.

### 3.3 Speciation of Silver in Tissues and Feces by LA-SP-ICP-MS

Since ICP-MS and LA-ICP-MS only provide information about total silver regardless of its physicochemical form, LA-ICP-MS operating in single particle mode was applied to determine if silver was present in ionic or particulate forms in the solid samples. The experimental conditions for the detection of silver nanoparticles by LA-ICP-MS in single particle mode were based on those optimized by Metarapi et al.^33^ The fluence was maintained at 1 J cm^–2^, whereas the laser beam size was increased to 20 μm using sample slices of 5 μm thickness.

No particles containing silver were detected in the liver tissues of pigs fed with 2% of additive, suggesting that the silver detected by LA-ICP-MS (Figure 1) was in ionic form, in agreement with in vitro gastrointestinal digestion studies, where ionic silver was the main species present in the bioaccessible fraction.^47^ Figure 3 shows the distribution of particulate silver and ionic silver in pig feces corresponding to the P2 group, showing a homogeneous distribution of both silver forms. The intensity signals corresponding to the particles were converted into particle size, expressed as the equivalent diameter of spherical nanoparticles containing the mass of silver detected. The silver-containing particles detected in pig feces showed a distribution with the most frequent equivalent diameter of 50 nm, corresponding to particles containing 520 ag of silver (Figure 4). Chicken feces corresponding to the C2 treatment were also analyzed using the same conditions as for pig feces, although a low number of particles, as well as ionic silver, were detected (Figure S3 of the SI), concluding that the Ag–kaolin administrated to chickens should be excreted during the removal period.

**Figure 3:**
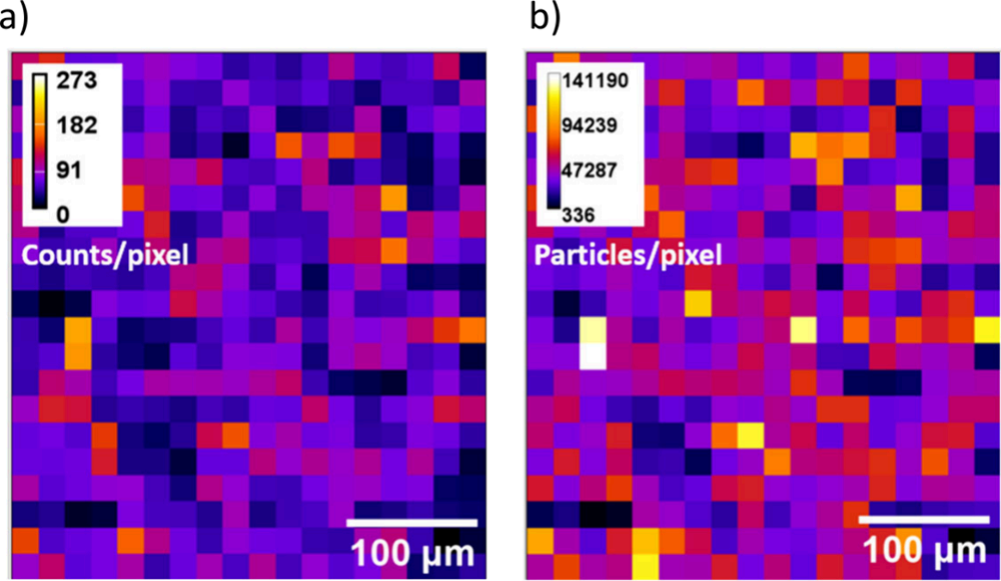
Ag-containing particles (a) and Ag­(I) (b) mappings of pig feces from P2 group measured by LA-SP-ICP-MS.

**Figure 4:**
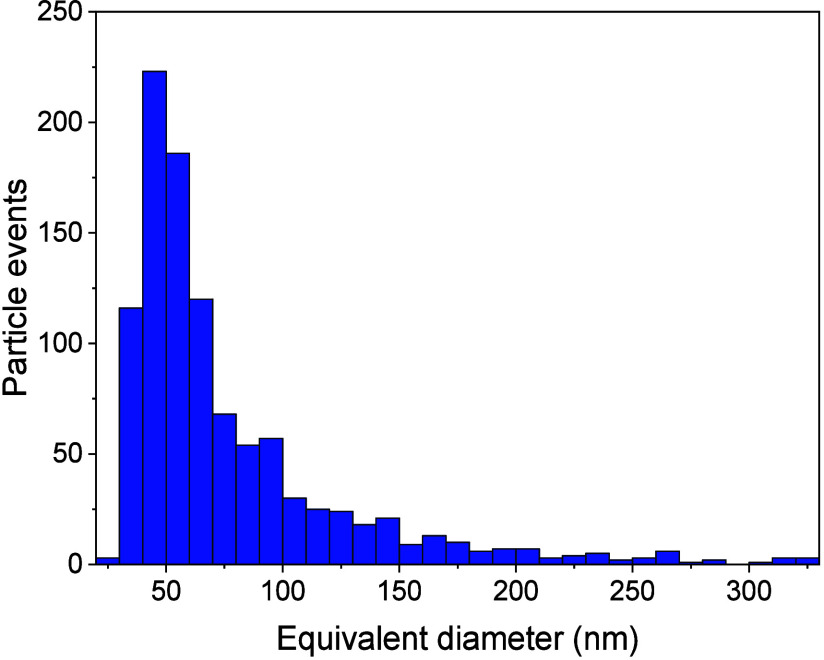
Size distribution of Ag-containing particles found in pig feces expressed as equivalent diameters and measured by LA-SP-ICP-MS

In this study, the administration of a low concentration of the nanomaterial Ag–kaolin (up to 2% (m/m) of additive in feed) to animals did not cause accumulation of silver in muscles, while a small level of the element was detected in the liver of chickens and pigs. On the other hand, silver was mainly excreted through feces, confirming the elimination of silver via the digestive tract. Imaging analysis by LA-ICP-MS confirmed the accumulation of silver in the outer layer of the lobule of the liver in pigs fed with high additive levels of 2% (m/m). Analysis by LA-SP-ICP-MS allowed differentiation of the presence of ionic and particulate species of silver. Silver was present in pig liver in ionic form, whereas ionic and particles containing silver were detected in feces, although for low doses of additive (0.2%), only the presence of ionic forms could be confirmed. In summary, the absence of silver in muscle tissues of pig and chickens at low and high doses of the supplemented Ag–kaolin makes the additive a potential alternative as a growth promoter in animal feeding.

## Supplementary Material



## Notes

Collected acid-digested samples, samples analyzed by LA-SP-ICP-MS, parameters for total silver determination, AgNP sizing of LA-SP-ICP-MS line scan data, imaging optimization, and AgNPs and Ag­(I) maps (PDF)

## Abbreviations

Ag-kaolin: silver-kaolin
AgNPs: silver nanoparticles
LA-SP-ICP-MS: laser ablation single particle inductively coupled plasma

## References

[1] OECD-FAO . OECD-FAO Agricultural Outlook 2021–2030; July 5, 2021.

[2] Brown E. E. F., Cooper A., Carrillo C., Blais B. (2019). Selection
of Multidrug-Resistant
Bacteria in Medicated Animal Feeds. Front. Microbiol..

[3] Holmer I., Salomonsen C. M., Jorsal S. E., Astrup L. B., Jensen V. F., Høg B. B., Pedersen K. (2019). Antibiotic Resistance in Porcine
Pathogenic Bacteria and Relation to Antibiotic Usage. BMC Vet Res..

[4] Yang Y., Ashworth A. J., Willett C., Cook K., Upadhyay A., Owens P. R., Ricke S. C., DeBruyn J. M., Moore P. A. (2019). Review
of Antibiotic Resistance, Ecology, Dissemination, and Mitigation in
U.S. Broiler Poultry Systems. Front. Microbiol..

[5] Roth, N. ; Käsbohrer, A. ; Mayrhofer, S. ; Zitz, U. ; Hofacre, C. ; Domig, K. J. The Application of Antibiotics in Broiler Production and the Resulting Antibiotic Resistance in Escherichia Coli: A Global Overview. Poultry Science; Oxford University Press, 2019; pp 1791–1804. 10.3382/ps/pey539. PMC6414035 30544256

[6] Regulation (EC) No 1831/2003 of the European Parliament and of the Council of 22 September 2003 on Additives for Use in Animal Nutrition; European Commission, 2003; pp 29–43.

[7] Durán N., Durán M., de Jesus M. B., Seabra A. B., Fávaro W. J., Nakazato G. (2016). Silver Nanoparticles: A New View on Mechanistic Aspects
on Antimicrobial Activity. Nanomedicine.

[8] Zheng K., Setyawati M. I., Leong D. T., Xie J. (2018). Antimicrobial Silver
Nanomaterials. Coord. Chem. Rev..

[9] Zorraquín-Peña I., Cueva C., Bartolomé B., Moreno-Arribas M. V. (2020). Silver
Nanoparticles against Foodborne Bacteria. Effects at Intestinal Level
and Health Limitations. Microorganisms.

[10] Tammam A. M., Ibrahim S. A., Hemid A. A., Abdel-Azeem F., El-Faham A. I., Ali N. G. M., Salem W. (2020). Effect of
Nanoparticles
Supplementation in Broiler Diets on Performance, Microbial Population
and Digestive Tract Measurements. Int. J. Vet
Sci..

[11] Tammam A., Ibrahim S., Hemid A., Abdel-Azeem F., El-Faham A., Ali N., Salem W. (2021). Effect of
Silver Nanoparticles
As a Water Supplementation on Productive Performance, Carcass Characteristics
and Bone Measurements of Broiler Chicks. Egypt.
J. Nutr. Feeds.

[12] Al-Sultan S. I., Hereba A. R. T., Hassanein K. M. A., Abd-Allah S. M. S., Mahmoud U. T., Abdel-Raheem S. M. (2022). The Impact
of Dietary Inclusion of
Silver Nanoparticles on Growth Performance, Intestinal Morphology,
Caecal Microflora, Carcass Traits and Blood Parameters of Broiler
Chickens. Ital. J. Anim. Sci..

[13] Salem H. M., Ismael E., Shaalan M. (2021). Evaluation
of the Effects of Silver
Nanoparticles against Experimentally Induced Necrotic Enteritis in
Broiler Chickens. Int. J. Nanomed..

[14] Awaad M. H. H., Moustafa K. M. El., Zoulfakar S. A., Elhalawany M. S., Mohammed F. F., El-Refay R. M., Morsy E. A. (2021). The Role
of Silver Nanoparticles in the Reluctance of Colisepticemia in Broiler
Chickens. J. Appl. Poultry Res..

[15] Gallocchio F., Biancotto G., Cibin V., Losasso C., Belluco S., Peters R., Van Bemmel G., Cascio C., Weigel S., Tromp P., Gobbo F., Catania S., Ricci A. (2017). Transfer Study
of Silver Nanoparticles in Poultry Production. J. Agric. Food Chem..

[16] Dosoky W. M., Fouda M. M. G., Alwan A. B., Abdelsalam N. R., Taha A. E., Ghareeb R. Y., El-Aassar M. R., Khafaga A. F. (2021). Dietary Supplementation of Silver-Silica Nanoparticles
Promotes Histological, Immunological, Ultrastructural, and Performance
Parameters of Broiler Chickens. Sci. Rep..

[17] Thi
Ngoc Dung T., Nang Nam V., Thi Nhan T., Ngoc T. T. B., Minh L. Q., Nga B. T. T., Phan Le V., Viet Quang D. (2019). Silver Nanoparticles
as Potential Antiviral Agents against African Swine Fever Virus. Mater. Res. Express.

[18] Abad-Álvaro I., Trujillo C., Bolea E., Laborda F., Fondevila M., Latorre M. A., Castillo J. R. (2019). Silver
Nanoparticles-Clays Nanocomposites
as Feed Additives: Characterization of Silver Species Released during
in Vitro Digestions. Effects on Silver Retention in Pigs. Microchem. J..

[19] Fondevila M., Herrer R., Casallas M. C., Abecia L., Ducha J. J. (2009). Silver
Nanoparticles as a Potential Antimicrobial Additive for Weaned Pigs. Anim. Feed Sci. Technol..

[20] Fondevila, M. Potential Use of Silver Nanoparticles as an Additive in Animal Feeding. In Silver Nanoparticles; InTech, 2010; pp 325–334. 10.5772/8509.

[21] Saadh M. (2022). Potent Antiviral
Effect of Green Synthesis Silver Nanoparticles on Newcastle Disease
Virus. Arabian J. Chem..

[22] Ahmed H. A., El Bayomi R. M., Hamed R. I., Mohsen R. A., El-Gohary F. A., Hefny A. A., Elkhawaga E., Tolba H. M. N. (2022). Genetic Relatedness,
Antibiotic Resistance, and Effect of Silver Nanoparticle on Biofilm
Formation by Clostridium Perfringens Isolated from Chickens, Pigeons,
Camels, and Human Consumers. Vet Sci..

[23] Losasso C., Belluco S., Cibin V., Zavagnin P., MiÄetiÄ‡ I., Gallocchio F., Zanella M., Bregoli L., Biancotto G., Ricci A. (2014). Antibacterial Activity of Silver Nanoparticles: Sensitivity of Different
Salmonella Serovars. Front. Microbiol..

[24] Saleh A. A., El-Magd M. A. (2018). Beneficial Effects
of Dietary Silver Nanoparticles
and Silver Nitrate on Broiler Nutrition. Environ.
Sci. Pollut. Res..

[25] El-Abd N. M., Hamouda R. A., Al-Shaikh T. M., Abdel-Hamid M. S. (2022). Influence
of Biosynthesized Silver Nanoparticles Using Red Alga Corallina Elongata
on Broiler Chicks’ Performance. Green
Process. Synth..

[26] Kumar I., Bhattacharya J., Das B. K. (2020). Dispersion, Availability, and Antimicrobial
Activity of Silver Nanoparticles during Application to Drinking Water
of the Poultry. Environ. Nanotechnol. Monit.
Manage..

[27] Kumar I., Bhattacharya J. (2019). Assessment of the Role of Silver Nanoparticles in Reducing
Poultry Mortality, Risk and Economic Benefits. Appl. Nanosci. (Switzerland).

[28] More S., Bampidis V., Benford D., Bragard C., Halldorsson T., Hernández-Jerez A., Hougaard Bennekou S., Koutsoumanis K., Lambré C., Machera K., Naegeli H., Nielsen S., Schlatter J., Schrenk D., Silano V., Turck D., Younes M., Castenmiller J., Chaudhry Q., Cubadda F., Franz R., Gott D., Mast J., Mortensen A., Oomen A. G., Weigel S., Barthelemy E., Rincon A., Tarazona J., Schoonjans R. (2021). Guidance on
Risk Assessment of Nanomaterials to Be Applied in the Food and Feed
Chain: Human and Animal Health. EFSA J..

[29] Ahmadi F., Ahmadi F., Rahimi F. (2011). The Effect
of Different Levels of
Nano on Performances and Retention of Silver in Edible Tissues of
broilers. World Appl. Sci. J..

[30] Chauke, N. ; Siebrits, F. Evaluation of Silver Nanoparticles as a Possible Coccidiostat in Broiler Production. S. Afr. J. Anim. Sci. 2012, 42 (5). 10.4314/sajas.v42i5.10.

[31] Doble P. A., de Vega R. G., Bishop D. P., Hare D. J., Clases D. (2021). Laser Ablation-Inductively
Coupled Plasma-Mass Spectrometry Imaging in Biology. Chem. Rev..

[32] Metarapi D., Van Elteren J. T. (2020). Fundamentals
of Single Particle Analysis in Biomatrices
by Laser Ablation-Inductively Coupled Plasma Mass Spectrometry. J. Anal At Spectrom.

[33] Metarapi D., Van Elteren J. T., Šala M., Vogel-Mikuš K., Arčon I., Šelih V. S., Kolar M., Hočevar S. B. (2021). Laser Ablation-Single-Particle-Inductively
Coupled Plasma Mass Spectrometry as a Multimodality Bioimaging Tool
in Nano-Based Omics. Environ. Sci. Nano.

[34] Metarapi D., Šala M., Vogel-Mikuš K., Šelih V. S., Van Elteren J. T. (2019). Nanoparticle
Analysis in Biomaterials Using Laser Ablation-Single
Particle-Inductively Coupled Plasma Mass Spectrometry. Anal. Chem..

[35] Wang M., Zheng L., Wang B., Yang P., Fang H., Liang S., Chen W., Feng W. (2022). Laser Ablation-Single
Particle-Inductively Coupled Plasma Mass Spectrometry as a Sensitive
Tool for Bioimaging of Silver Nanoparticles in Vivo Degradation. Chin. Chem. Lett..

[36] Yamashita S., Ogawa K., Hirata T. (2021). Quantitative
Imaging Analysis of
Nanoparticles and Dissolved Forms Using Laser Ablation-Single Particle-ICP-Mass
Spectrometry. Metallomics Res..

[37] Nordhorn I. D., Dietrich D., Verlemann C., Vennemann A., Schmid R., Elinkmann M., Fuchs J., Sperling M., Wiemann M., Karst U. (2021). Spatially
and Size-Resolved Analysis
of Gold Nanoparticles in Rat Spleen after Intratracheal Instillation
by Laser Ablation-Inductively Coupled Plasma-Mass Spectrometry. Metallomics.

[38] Rodriguez-Garraus A., Azqueta A., Laborda F., Gimenez-Ingalaturre A. C., Ezquerra A., Lostao L., Lopez de Cerain A. (2022). In Vitro Genotoxicity
Evaluation of an Antiseptic Formulation Containing Kaolin and Silver
Nanoparticles. Nanomaterials.

[39] Pérez-Etayo L., González D., Leiva J., Díez-Leturia M., Ezquerra A., Lostao L., Vitas A. I. (2021). Antibacterial Activity
of Kaolin–Silver Nanomaterials: Alternative Approach to the
Use of Antibiotics in Animal Production. Antibiotics.

[40] Zaoui Y., Belanche A., Ben-Jeddou K., Jiménez M. S., Fondevila G., Fondevila M. (2024). Effect of
the Dietary Administration
Pattern of Silver Nanoparticles on Growth Performance, Biodiversity
of Digestive Microbiota and Tissue Retention in Broiler Chickens. Anim Feed Sci. Technol..

[41] Šala M., Šelih V. S., Van Elteren J. T. (2017). Gelatin Gels as Multi-Element Calibration
Standards in LA-ICP-MS Bioimaging: Fabrication of Homogeneous Standards
and Microhomogeneity Testing. Analyst.

[42] Schweikert A., Theiner S., Šala M., Vician P., Berger W., Keppler B. K., Koellensperger G. (2022). Quantification in Bioimaging by LA-ICPMS
- Evaluation of Isotope Dilution and Standard Addition Enabled by
Micro-Droplets. Anal. Chim. Acta.

[43] Jiménez-Lamana J., Laborda F., Bolea E., Abad-Álvaro I., Castillo J. R., Bianga J., He M., Bierla K., Mounicou S., Ouerdane L., Gaillet S., Rouanet J. M., Szpunar J. (2014). An Insight into Silver Nanoparticles
Bioavailability
in Rats. Metallomics.

[44] van
der Zande M., Vandebriel R. J., Van Doren E., Kramer E., Herrera Rivera Z., Serrano-Rojero C. S., Gremmer E. R., Mast J., Peters R. J. B., Hollman P. C. H., Hendriksen P. J. M., Marvin H. J. P., Peijnenburg A. a C. M., Bouwmeester H. (2012). Distribution, Elimination, and Toxicity of Silver Nanoparticles
and Silver Ions in Rats after 28-Day Oral Exposure. ACS Nano.

[45] van
Elteren J. T., Šala M., Metarapi D. (2021). Comparison of Single
Pulse, Multiple Dosage, and 2D Oversampling/Deconvolution LA-ICPMS
Strategies for Mapping of (Ultra)­Low-Concentration Samples. Talanta.

[46] Ricken T., Werner D., Holzhütter H.
G., König M., Dahmen U., Dirsch O. (2015). Modeling Function–Perfusion
Behavior in Liver Lobules Including Tissue, Blood, Glucose, Lactate
and Glycogen by Use of a Coupled Two-Scale PDE–ODE Approach. Biomech Model Mechanobiol.

[47] Ben-Jeddou K., Bakir M., Jimenez M. S., Gomez M. T., Abad-Alvaro I., Laborda F. (2024). Nanosilver-Based Materials
as Feed Additives: Evaluation
of Their Transformations along in vitro Gastrointestinal Digestions
in Pigs and Chickens by Using an ICP-MS Based Analytical Platform. Anal. Bioanal. Chem..

