# Supplementary material for: Evaluation of the Accumulation, Distribution, and Excretion of Different Silver Species in Tissues and Feces from Chickens and Pigs Fed with Silver-Based Nanomaterial Supplemented Feeds
Source: ACS Agric Sci Technol. 2025 Feb 25;5(4):454–60. doi: 10.1021/acsagscitech.4c00338 (PMC12127977; doi:10.1021/acsagscitech.4c00338)

Evaluation of the accumulation, distribution and excretion of different silver species in tissues and faeces from chickens and pigs fed with silver-based nanomaterial supplemented feeds

Khaoula Ben-Jeddou[a], Mariam Bakir[a], M. Sierra Jiménez[a], Manuel Fondevila[b], Dino Metarapi[c], Martin Šala[c], Johannes T. van Elteren[c], Francisco Laborda[a]

Supporting Information

List of items

Table S1. The acid-digested samples collected from the chicken and pig experiments

Table S2. Samples analysed by LA-SP-ICP-MS (3 samples for each treatment per animal)

Table S3. ICP-MS instrumental and data acquisition parameters for total silver determination

Figure S1. AgNP sizing of line scan data of LA-SP-ICP-MS

Figure S2. Optimization of LA-ICP-MS imaging parameters

Figure S3. a) AgNPs map of chicken's faeces (C2) (number concentration of particles per pixel), b) Ag(I) map of chicken's faeces (calibration bar in particles/pixel)

Table S1. The acid-digested samples collected from the chicken and pig experiments

	Chicken samples				Pig samples		
	**Control**	**C1**	**C2**	**C3**	**Control**	**P1**	**P2**
**Muscle**	24	12	12	12	12	17	18
**Liver**	24	12	12	12	12	17	18
**Kidney**	-	-	-	-	12	17	17
**Faeces**	24	8	3	3	7	8	8

Table S2. Samples analysed by LA-SP-ICP-MS (3 samples for each treatment per animal)

	**chickens**	**Pigs**
**Liver**	Control	Control
	C1	P2
**Faeces**	Control	Control
	C2	P1
		P2

(The C1 and C3 samples of chicken faeces with the highest content of silver were no longer available for LA analysis)

Table S3. ICP-MS instrumental and data acquisition parameters for total silver determination

Instrumental parameters	
RF power	
Argon flow rate	
Plasma	15 L min^{-1}
Auxiliary	1.2 L min^{-1}
Nebulizer	1.04 L min^{-1}
Sample flow rate	0.3 mL min^{-1}
Data acquisition parameters	
Dwell time	50 ms
Number of readings	20
Readings per replicate	1
Number of replicates	10
Settling time	0 ms
Total acquisition time	30 s
Isotopes monitored	^{107}Ag, ^{103}Rh

Figure S1. AgNP sizing of line scan data of LA-SP-ICP-MS

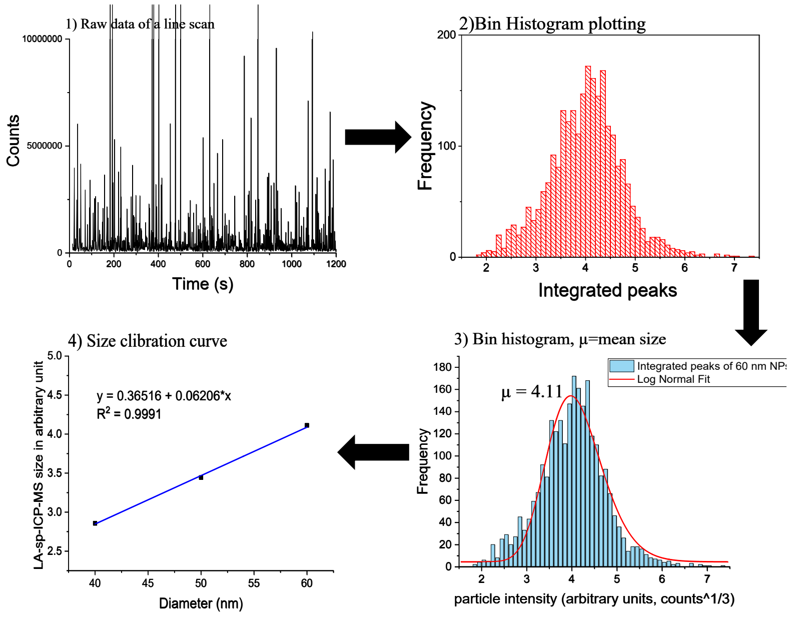

Figure S2. Optimization of LA-ICP-MS imaging parameters

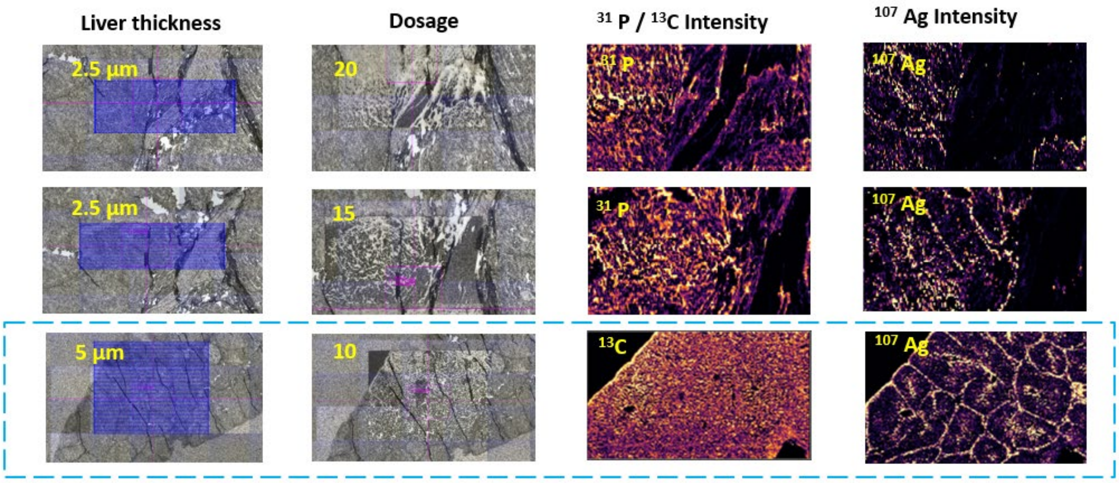

Figure S3. a) AgNPs map of chicken's faeces (number of particles/pixel), b) Ag(I) map of chicken's faeces (calibration bar in counts/pixel)

a) b)

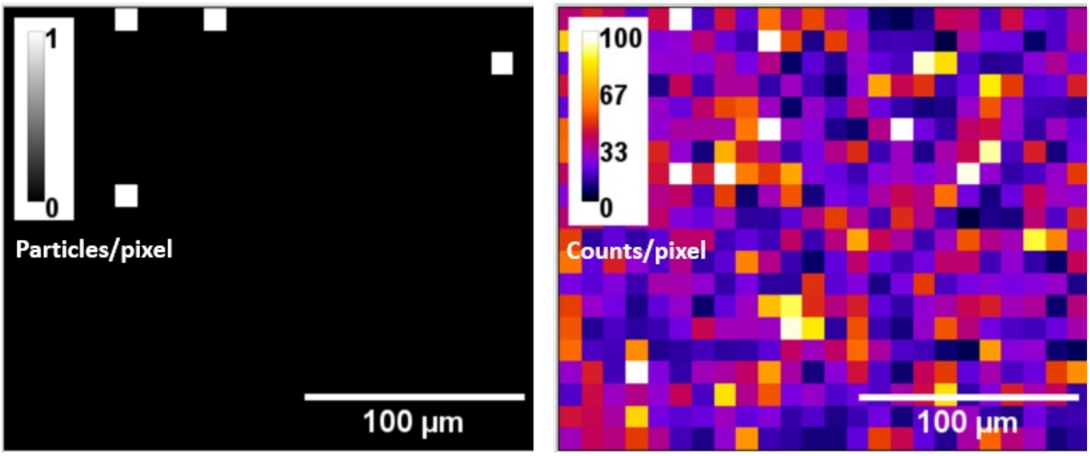

Supplement: Supplementary file 1 [file as4c00338_si_001.pdf]
